# Rate, determinants, and causes of stillbirth in Jordan: Findings from the Jordan Stillbirth and Neonatal Deaths Surveillance (JSANDS) system

**DOI:** 10.1186/s12884-020-03267-2

**Published:** 2020-09-29

**Authors:** Khulood K. Shattnawi, Yousef S. Khader, Mohammad S. Alyahya, Nihaya Al-Sheyab, Anwar Batieha

**Affiliations:** 1 Maternal & Child Health Nursing Department, Faculty of Nursing, Jordan University of Science and Technology, P.O.Box (3030, 22110 Irbid, Jordan; 2 Department of Community Medicine, Public Health and Family Medicine, Faculty of Medicine, Jordan University of Science & Technology, 22110 Irbid, Jordan; 3 Department of Health Management and Policy, Faculty of Medicine, Jordan University of Science and Technology, P.O. Box 3030, 22110 Irbid, Jordan; 4 Allied Medical Sciences Department/Faculty of Applied Medical Sciences, the Faculty of Nursing, Jordan University of Science and Technology, P.O. Box 3030, 22110 Irbid, Jordan; 5 Department of Public Health, Jordan University of Science and Technology, Irbid, Jordan

**Keywords:** Stillbirth, Jordan, Rate, Determinants, Surveillance

## Abstract

**Background:**

Annually, 2.6 million stillbirths occur around the world, with approximately 98% occurring in low- and middle-income countries. The stillbirth rates in these countries are 10 times higher than the rates in high-income countries.

**Methods:**

An electronic stillbirths and neonatal deaths surveillance system (JSANDS) was established in five large hospitals located in three of the largest cities in Jordan in August 2019. JSANDS was developed as a secure on-line data entry system to collect, organize, analyze, and disseminate data on stillbirths, neonatal deaths, and their contributing conditions. Data on births, stillbirths and their contributing conditions, and other demographic and clinical characteristics in the period between August 2019 – January 2020 were extracted and analyzed.

**Results:**

A total of 10,328 births were registered during the reporting period. Of the total births, 102 were born dead (88 antepartum stillbirths and 14 intrapartum stillbirths), with a rate of 9.9 per 1000 total births. The main contributing fetal conditions of antepartum stillbirths were antepartum death of unspecified cause (33.7%), acute antepartum event (hypoxia) (33.7%), congenital malformations and chromosomal abnormalities (13.3%), and disorders related to the length of gestation and fetal growth (10.8%). The main contributing maternal conditions of antepartum stillbirths included complications of the placental cord and membranes (48.7%), maternal complications of pregnancy (23.1%), and maternal medical and surgical conditions (23.1%). Contributing fetal conditions of intrapartum stillbirths included congenital malformations, deformations and chromosomal abnormalities, other specified intrapartum disorders, and intrapartum death of unspecified cause (33.3% each). Contributing maternal conditions of intrapartum stillbirths included complications of the placental cord and membranes. In the multivariate analysis, small for gestational age (SGA) pregnancies were associated with a significant 3-fold increased risk of stillbirth compared to appropriate for gestational age (AGA) pregnancies.

**Conclusions:**

Although the rate of stillbirth is lower than that in other countries in the region, there is an opportunity to prevent such deaths. While the majority of stillbirths occurred during the antepartum period, care should be taken for the early identification of high-risk pregnancies, including the early detection of SGA pregnancies, and ensuring adequate antenatal obstetric interventions.

## Background

Stillbirth is considered a global public health problem, particularly in developing countries. The global burden of disease study reported a 47.0% decline in stillbirth rates between 1990 and 2015 [1]. Data extracted from registration systems from 157 countries reported that an estimate of 2.6 million stillbirths occurred annually and showed a reduction of 25.5% in stillbirth rates for the period 2000 until 2015 [2]. Approximately 98% of these stillbirths occurred in low- and middle-income countries [3].

Regionally, a retrospective population-based study of stillbirth in a multi-ethnic Middle-Eastern population reported a stillbirth rate of 7.81 per 1000 total births [4]. The majority of stillbirths in the developing countries occur unexpectedly, without a clear cause [5]. It is difficult to confirm the cause because there are many factors that may contribute to the cause of a stillbirth; however, the literature has categorized contributing conditions into those related to maternal or fetal conditions [6]. Maducolil et al. reported that maternal factors comprised a 52.4% of total stillbirths in the Middle-Eastern population and included maternal hypertension, diabetes, and other medical disorders. The main fetal factors reported in the same study were intrauterine growth restriction followed by congenital anomalies [4].

One study in Jordan reported the stillbirth rate during the period 2011–2012 and showed a stillbirth rate of 10.6 per 1000 total births [7]. This study reported maternal diseases, unexplained immaturity, congenital anomalies, unexplained antepartum stillbirths, obstetric complications, placental conditions, and multiple births as the main contributing conditions of stillbirths.

The scarcity of data in Jordan on stillbirth is generally linked to the fact that stillbirths are not registered [8]. In addition, the existing sources of data on stillbirths are liable to biases. Therefore, improving a reporting system of stillbirth and neonatal deaths is critical for tracking progress and taking appropriate actions. As a result of this limitation, an electronic stillbirths and neonatal deaths surveillance system (JSANDS) was developed and established in five large hospitals in Jordan in August 2019. JSANDS was developed as a secure on-line data entry system to collect, organize, analyze, and disseminate reliable data on stillbirths, neonatal deaths, and their contributing conditions. In addition, the system registers births to use them as a denominator for mortality measures. The definitions of the stillbirths and neonatal deaths used in the system were based on the international standards set by the WHO and the CDC. To ensure comparability of mortality rates between different providers and to allow for international comparisons, births less than 24^+ 0^ weeks of gestation and terminations of pregnancy are not reported by JSANDS. This study used the data from JSANDS to determine the rate, determinants, and contributing conditions of stillbirths in Jordan.

## Methods

This manuscript is reporting the results of the first phase of a larger study. The information on all deliveries and birth outcomes that were registered in JSANDS during the period from August 2019 to January 2020 was retrieved from the system. A team of researchers was assigned, one for each hospital, to monitor the delivery entries. As a result, all births and stillbirths that occurred in the five large hospitals were registered with a completeness of 100%. Three hospitals were public hospitals, one was private, and one was a teaching hospital. The extracted data included sociodemographic characteristics of both parents, delivery information (i.e., mode of delivery, multiplicity, and gestational age), newborn information (status, birth weight, Apgar score) and contributing conditions of stillbirths.

In this study, stillbirth was defined as any fetal death that occurred at or after 24 gestational weeks. Stillbirths were categorized to antepartum (deaths occurring prior to labor) and intrapartum stillbirths (deaths that occur after the onset of labor but prior to birth). The stillbirth rate was calculated as the number of stillbirths per 1000 total live births and stillborn births.

Contributing conditions of stillbirths were determined based on the International Classification of Diseases-Perinatal Mortality (ICD-PM), which is derived from the 10th version of the International Classification of Diseases (ICD-10) developed by the WHO [9]. ICD-PM is a standardized classification for reporting perinatal deaths [9]. All health care professionals in the five hospitals were trained on how to assign the main contributing condition of death. The doctor (an obstetrician) who was responsible for the delivery of a stillbirth accompanied by a neonatologist who attended the delivery had the primary responsibility to complete the form for the stillbirth, assign the main contributing condition of death, and record the ICD-10 code accordingly. ICD-10 codes were used to provide a common language for reporting and monitoring diseases. This allows for comparing and sharing data in a consistent and standard manner between the 5 hospitals.

Contributing conditions of deaths related to fetal or maternal condition were registered. First, the main disease or condition in the fetus was determined, in which the single most important and main disease or condition of the fetus who died was entered. This was supported by reporting any other diseases or conditions in the fetus, if any. As there is no uniform protocol to evaluate and classify stillbirths in Jordan, malformations and chromosomal abnormalities were determined based on the attending obstetrician’s and neonatologist’s judgments of the obvious stillbirth malformations. According to the hospital’s protocol for placental examination, visual examinations of the placentas and umbilical cords were performed. In addition, a detailed summary of every stillbirth was discussed in monthly death review committee meetings attended by obstetricians, neonatologists, and senior midwives and nurses. Second, the main maternal disease or condition affecting the fetus was reported. The “most important” maternal disease or condition affecting the fetus that made the greatest contribution to the fetal death was reported. Other maternal diseases or conditions affecting fetus, if any, were also reported in this section.

Data were described and analyzed using IBM SPSS version 24 (IBM Corp. Released 2016. IBM SPSS Statistics for Windows, Version 24.0. Armonk, NY: IBM Corp.). Data were described using means and standard deviations for continuous variables and rates and percentages for categorical data. The stillbirth rate was calculated as the number of stillbirths by 1000 total births. Fetal weight percentile was determined based on birth weight and fetal age for stillbirths. Fetal growth was categorized as small for gestational age (SGA) if < 10th percentile, appropriate for gestation if between 10th – 90th percentile, or large for gestation if > 90th percentile. The distributions of stillbirths according to studied characteristics, including birthweight for gestational age percentiles, were tested using the Chi-square test. Multivariate analysis using binary logistic regression was used to determine factors associated with stillbirth. A *p*-value of less than 0.05 was considered statistically significant.

The study was ethically approved by the Institutional Review Board (IRB) at Jordan University of Science and Technology (Ethical approval number 20,170,033). Permission from the Ministry of Health was received to access the data and use it for research purposes. To ensure the data confidentiality, the data were exported without identifying information, such as the name or phone number.

## Results

### Women’s demographic and maternal characteristics

During the period from August 2019 to January 2020, a total of 9983 women gave birth to 10,328 babies. The women’s ages ranged between 15 and 48 years, with a mean (SD) of 29.1 (6.1) years. The majority of women (81.0%) were between 19 and 35 years of age, 2.5% were younger than 19 years and 16.5% were older than 35 years. Table 1 shows the sociodemographic and maternal characteristics of the women.

**Table 1:** The sociodemographic and maternal characteristics of the women in the study (n = 9983)

Variables	%
**Hospital**	
Private	17.6
Teaching	13.7
Ministry of Health	68.7
**Mother age (year)**	
≤18	2.5
19–35	81.0
>35	16.5
**Mother Education Level**	
High school or less	55.4
Diploma	6.9
Bachelor	24.2
Master or higher	2.5
Unknown	11.0
**Total Income (JD)**	
<500	74.0
500-<1000	16.7
≥1000	2.1
Unknown	7.2
**Working status**	
Housewife	89.6
Employed	10.4
**Mode of delivery**	
Vaginal	51.2
Planned CS	27.2
Emergency CS	21.6
**Multiplicity**	
Single	97.4
Twin	2.4
Triplet	0.2
Quadruplet	0.0
**Gestational age**	
Full-term	90.3
Preterm	9.7

### Stillbirth rate

Of the total 10,328 births, 102 were stillborn (88 antepartum stillbirths and 14 intrapartum stillbirths). The overall stillbirth rate was 9.9 per 1000 total births. Table 2 shows the stillbirth rate according to the maternal, clinical and relevant characteristics. The stillbirth rate did not vary significantly according to health sector and mother’s age, educational level, income, and working status. However, the rate varied significantly according to multiplicity, birthweight and gestational age. The rate per 1000 total births was significantly higher in multiple births than in single births (8.7 in single births, 24.1 in twins, 69.8 in triplets, and 375.0 in quadruplets). The stillbirth rate was much higher among small for gestational age (SGA) deliveries compared to appropriate for gestational age (AGA) deliveries.

**Table 2:** Stillbirth rate according to the sociodemographic, maternal, clinical and relevant characteristics of the women and the birth characteristics

Variables	Total births (%)	Number of Stillbirths	Stillbirth rate per 1000 total births	*p*-value
**Hospital**				0.928
Private	1814 (17.6)	19	10.5	
Teaching	1447 (14.0)	15	10.4	
Ministry of Health	7067 (68.4)	68	9.6	
**Mother age (year)**				0.275
≤18	255 (2.5)	2	7.8	
19–35	8377 (81.1)	89	10.6	
>35	1696 (16.4)	11	6.5	
**Mother Education Level**				0.109
High school or less	5702 (55.2)	59	10.3	
Diploma	711 (6.9)	2	2.8	
Bachelor	2502 (24.2)	26	10.4	
Master or higher	259 (2.5)	0	0.0	
Unknown	1154 (11.2)	15	13.0	
**Total Income (JD)**				0.905
<500	7633 (73.9)	75	9.8	
500-<1000	1718 (16.6)	19	11.1	
≥1000	217 (2.1)	2	9.2	
Unknown	760 (7.4)	6	7.9	
**Working status**				0.869
Housewife	9265 (89.7)	91	9.8	
Employed	1063 (10.3)	11	10.3	
**Multiplicity**				< 0.001
Single	9777 (95.1)	85	8.7	
Twin	457 (4.4)	11	24.1	
Triplet	43 (0.4)	3	69.8	
Quadruplet	8 (0.1)	3	375.0	
**Birthweight for gestational age**				< 0.001
<10th Percentile	952 (9.2)	25	26.3	
10th – 90th Percentile	8304 (80.4)	70	8.4	
>90th Percentile	1072 (10.4)	7	6.5	
**Gender of baby**				0.465
Female	4791 (46.4)	51	10.6	
Male	5533 (53.6)	51	9.2	

### Determinants of stillbirths

Of all studied factors, only birthweight for gestational age percentiles was significantly associated with the risk of stillbirth (Table 3). SGA pregnancies were associated with a significant 3-fold increased risk of stillbirth compared to AGA pregnancies.

**Table 3:** The association between birthweight for gestational age percentiles and stillbirth

	OR	95% Confidence Interval		***p***-value
Birthweight for gestational age				
<10th Percentile	3.2	2.0	5.0	< 0.001
10th – 90th Percentile	Reference			
>90th Percentile	0.8	0.4	1.7	0.518

### Contributing conditions of stillbirths

Table 4 shows the main contributing conditions of antepartum stillbirths. The main leading contributing condition of antepartum stillbirths was an acute antepartum event (hypoxia), which contributed to 33.7% of antepartum stillbirths. One-third (33.7%) of antepartum stillbirths had no specific cause. The second and third contributing conditions of antepartum stillbirths were congenital malformations and chromosomal abnormalities (13.3%) and disorders related to length of gestation and fetal growth (10.8%), respectively. Maternal conditions contributed to 39 (47.0%) antepartum stillbirths. Complications of placenta cord and membranes contributed to 22.9%, maternal complications of pregnancy contributed to 10.8%, and maternal medical and surgical conditions contributed to 10.8% of antepartum stillbirths.

**Table 4:** Main contributing conditions of antepartum stillbirths

Contributing fetal conditions	N (%)
A1-Congenital malformations and chromosomal abnormalities	11 (13.3%)
A2-Infection	2 (2.4%)
A3-Acute antepartum event (hypoxia)	28 (33.7%)
A4-Other specified antepartum disorder	5 (6.0%)
A5-Disorders related to length of gestation and fetal growth	9 (10.8%)
A6-Antepartum death of unspecified cause	28 (33.7%)
Total	83 (100.0%)
**Contributing maternal conditions**	
M1-Complications of placenta cord and membranes	19 (22.9%)
M2-Maternal complications of pregnancy	9 (10.8%)
M3-Other complications of labor and delivery	2 (2.4%)
M4-Maternal medical and surgical conditions	9 (10.8%)
No maternal cause	44 (53.0%)

Table 5 shows the main contributing conditions of intrapartum stillbirths. Congenital malformations, deformations and chromosomal abnormalities contributed to 33.3% of intrapartum stillbirths, and other specified intrapartum disorders contributed to 33.3% of intrapartum stillbirths. The remaining intrapartum stillbirths had no specified cause. Complications of placenta cord and membranes contributed to 33.3% of intrapartum stillbirths.

**Table 5:** **Main contributing conditions of intrapartum stillbirths**

Contributing fetal conditions	N (%)
I1-Congenital malformations, deformations and chromosomal abnormalities	4 (33.3%)
I5-Other specified intrapartum disorder	4 (33.3%)
I7-Intrapartum death of unspecified cause	4 (33.3%)
Total	12 (100%)
**Contributing maternal conditions**	
M1-Complications of placenta cord and membranes	4 (33.3%)
No maternal causes	8 (66.7%)

## Discussion

Stillbirth rates vary widely between countries. According to Frøen et al., stillbirths are not counted in 90 countries worldwide [10], making it difficult to estimate the true rates of stillbirth. In the current study, the incidence of stillbirth was found to be 9.9 per 1000 live births. This rate is similar to the rate in Lebanon for the year 2015 (9.9) [11]. The rate is higher than the rate of 7.81 per 1000 births that was reported in a multi-ethnic Middle-Eastern-based study [4] and the rates reported for the year 2015 for some other Arab countries, such as Libya (8.8 per 1000 live births), Oman (8.5 per 1000 live births), Qatar (5.8 per 1000 live births), and Kuwait (5.1 per 1000 live births) [11]. However, the rate was lower than the rate of 11.6 per 1000 live births that was reported in a previous Jordan study in 2012 [8] and the 2015 rates in other countries, such Syria (11.1), Saudia Arabia (13.9), Egypt (12.2), Iraq (15.5), and Jordan (10.5) [11]. This decline is promising and might be related to improvements in maternal health care services, yet there remains room for more improvement. Most of these stillbirths are preventable, and a greater decline in stillbirth rate is possible. To achieve lower rates, we need to address possible risk factors and possible contributing conditions of stillbirths.

Our study showed that SGA pregnancies and multiple gestations are risk factors of stillbirth. These are well-known risk factors in the literature and are closely linked to stillbirths [12–14]. SGA may result from both fetal growth restriction and preterm birth, which are associated with placental dysfunction and subsequent poor fetal outcomes [2]. This increases the risk of both antepartum and intrapartum fetal deaths [2]. As stillbirth rates are very sensitive to access to high-quality antenatal health care [6], proper assessment and early identification of multiple gestations, gestational age, and birth weight may contribute to the decrease of the incidence of stillbirths.

Although advanced maternal age of > 35 years was reported as a significant factor for increased stillbirth rate [15, 16], it was not found to be significant in the current study. The exact mechanism of the increase risk of stillbirth with advanced maternal age is not fully understood [17], which necessitates additional studies to determine the mechanism. However, some research suggested that advanced maternal age is associated with placental dysfunction, which may increase the risk of stillbirths [17], or to existing maternal medical conditions [18]. Nevertheless, of the existing evidence, the lack of relationship in this study between advanced maternal age and risk of stillbirth may be explained by the low percentage of mothers of advanced age in our sample.

Based on the WHO ICD-PM classification, the main two contributing fetal conditions of antepartum stillbirth in this study were antepartum death of unspecified cause and acute antepartum event (hypoxia), followed by congenital malformations and chromosomal abnormalities. Allanson et al. [19] have demonstrated the application of the WHO ICD-PM to perinatal deaths in two data sets from the UK and South Africa. Similar to our findings, they reported antepartum hypoxia events as the major causes of antepartum deaths for the South Africa data set. Maternal conditions that were associated with these deaths were mostly medical and surgical conditions. For the UK data set, the majority of antepartum deaths were unexplained deaths to healthy mothers. Antepartum hypoxia is one of the most significant problems contributing to stillbirths and neonatal deaths and can be caused by many conditions, such as placental insufficiency [20].

Congenital malformation was reported constantly across many classification systems [6], which could be preventable by the use of prenatal folic acid supplements, which have been proved to decrease the incidence of congenital abnormalities such as neural tube defects [21].

Complications of the placental cord and membranes were identified as the main contributing maternal conditions of stillbirth in our study. Previous studies showed that a significant percentage of stillbirths arises from placental problems [15, 22]. Because of the inadequate oxygen supply to the fetus, placental dysfunction is linked to intrauterine growth restriction, preterm birth, and birth defects [23], which significantly increase the perinatal mortality and morbidity. This explains why SGA pregnancies are considered the main contributing factors of stillbirths.

Intrapartum stillbirths were relatively few in our study; however, they were significant in highlighting a very important contributing condition of stillbirth. Fetal contributing conditions of the intrapartum stillbirths included congenital malformations deformations and chromosomal abnormalities, other specified intrapartum disorder, and intrapartum death of unspecified cause. Maternal contributing conditions of intrapartum stillbirths included complications of the placental cord and membranes. The majority of these deaths could be prevented with reasonably priced interventions [24]. Bhutta and others proposed a basic package of antenatal interventions to reduce the incidence of antepartum and intrapartum stillbirths. The package includes periconceptional folic acid supplementation, prevention of malaria, detection and management of syphilis during pregnancy, and basic and comprehensive emergency obstetric care [25]. Other interventions may include testing of high-risk pregnancies, ultrasonographic monitoring, and iatrogeneic deliveries [26]. As there is no clear evidence that ultrasonographic monitoring is harmful during pregnancy [27], it could be used with high-risk pregnancies for its many presumed benefits, including better estimation of gestational age, earlier detection of multiple pregnancies, placental abnormalities, fetal malformation and intrauterine fetal growth restriction [27].

The antepartum stillbirths reflect the quality that women receive during the antenatal period, while the intrapartum stillbirths reflect the quality of care that they receive during delivery [28]. The WHO has reported that deficiencies in the quality of antenatal care play a significant role in increasing the stillbirth rate [29]. Research has documented a significant decrease in stillbirths with higher quality antenatal care, women’s education, and regular antenatal visits, and recommended more involvements of the health care provider in teaching mothers about the danger signs of pregnancy rather than providing only the basic health care assessments, such as measuring their blood pressure [30].

Maternal mortality and stillbirth are strongly correlated. It is imperative, therefore, to increase our attention for stillbirths and preterm birth interventions, which will positively impact the maternal and newborn health outcomes [31]. Investing in the health care system and providing good-quality and timely maternal services may prevent a significant ratio of stillbirths [32]. In interpretation of the study findings, one should consider that the study of specific causes of stillbirth has been hampered by lack of investigations such as fetal autopsy and genetic evaluation.

## Conclusions

Although the rate of stillbirth is declining and is lower than that in other countries in the region, there is an opportunity to prevent such deaths. While the majority of stillbirths occurred during the antepartum period, care should be taken for the early identification of high-risk pregnancies, including the early detection of SGA pregnancies, and ensuring adequate antenatal obstetric interventions. SGA pregnancies and multiple gestations are reported as risk factors in the current study; therefore, efforts must be directed toward early prediction of these risks so that appropriate and timely interventions may be implemented to reduce stillbirths. This study reported the findings of stillbirths’ data extracted from a national neonatal and stillbirth surveillance system. As the majority of stillbirths and neonatal deaths are not reported in Jordan, investing in the health information systems to improve data registration will encourage appropriate use of interventions to reduce stillbirth rates.

## Data Availability

The datasets used and/or analyzed during the current study are available from the corresponding author on reasonable request.

## Abbreviations

JSANDS: Jordan Stillbirth and Neonatal Surveillance.
ICD-PM: International Classification of Diseases-Perinatal Mortality.
